# High prevalence of psychiatric comorbidities in children and adolescents at a tertiary epilepsy center

**DOI:** 10.1590/0004-282X-ANP-2020-0202

**Published:** 2021-06-23

**Authors:** Maria Antonia Serra-Pinheiro, Isabella D'andrea-Meira, Abraão Iuri Medeiros Angelim, Fernanda Alves Fonseca, Nicolle Zimmermann

**Affiliations:** 1 Instituto Estadual do Cérebro Paulo Niemeyer Rio de Janeiro RJ Brazil Instituto Estadual do Cérebro Paulo Niemeyer, Rio de Janeiro RJ, Brazil.; 2 Universidade Federal Fluminense Niterói RJ Brazil Universidade Federal Fluminense, Niterói RJ, Brazil.

**Keywords:** Epilepsy, Comorbidity, Children, Epilepsia, Comorbidade, Crianças

## Abstract

**Background:**

Epilepsy is highly comorbid with psychiatric disorders and a significant amount of the morbidity related to epilepsy is in fact a result of psychiatric comorbidities.

**Objective:**

To investigate the frequency of different psychiatric comorbidities in children with refractory epilepsy.

**Methods:**

We present preliminary observational data from a series of patients (n=82) examined in the psychiatric branch of a tertiary epilepsy center in Rio de Janeiro, Brazil. Patients were classified as presenting autism spectrum disorders, mood disorders, anxiety disorders, disruptive disorders, attention deficit hyperactivity disorder (ADHD), intellectual development disorder, psychotic episode, dissociative/conversive disorders or others. We determined the frequency of each disorder, along with demographic data, medications prescribed, electroencephalogram findings and additional medical examinations and consultations.

**Results:**

The most common comorbidities in our sample were autism spectrum disorders and ADHD. Antipsychotics and selective serotonin uptake inhibitors were the most commonly prescribed psychiatric medications.

**Conclusions:**

Knowledge about the prevalence of such comorbidities may provide more targeted interventions in Psychiatry and Psychology services linked to epilepsy centers.

## RESUMO

**Introdução:**

Epilepsia é altamente comórbida, com transtornos psiquiátricos, e uma parte significativa da morbidade da epilepsia se associa com os transtornos psiquiátricos comórbidos.

**Objetivo:**

Investigar a frequência de diferentes comorbidades psiquiátricas em crianças com epilepsia refratária.

**Métodos:**

Apresentamos dados observacionais preliminares de uma amostra de pacientes (n=82) avaliados no setor de Psiquiatria de um centro terciário de tratamento de epilepsia no Rio de Janeiro, Brasil. Pacientes foram classificados como apresentando transtorno do espectro autista, transtorno do humor, transtorno de ansiedade, transtornos disruptivos, transtorno do déficit de atenção de hiperatividade (TDAH), transtorno do desenvolvimento intelectual, episódio psicótico, transtornos dissociativos/conversivos e outros. Foram determinados frequência de cada transtorno, bem como dados demográficos, medicações prescritas e achados de eletroencefalograma.

**Resultados:**

As comorbidades mais comuns na nossa amostra foram transtornos do espectro autista e TDAH; antipsicóticos e inibidores seletivos da recaptura de serotonina (ISRS) foram as medicações psiquiátricas mais comumente prescritas.

**Conclusões:**

Conhecimento acerca da prevalência dessas comorbidades pode facilitar a instituição de intervenções mais precisas em serviços de Psiquiatria e Psicologia vinculados a centros de tratamento de epilepsia.

## INTRODUCTION

Epilepsy is a common condition worldwide that gives rise to a significant burden for patients. Among children, the estimates for the incidence of epilepsy vary widely, ranging from 41 to 187/100,000/year. A very significant subset of epileptic patients does not achieve remission with the treatments available. Rosati et al.^1^ estimated that 20% of epileptic children continue to have seizures despite their use of antiepileptic drugs.

Epilepsy is a highly comorbid condition. Clinical and psychiatric conditions co-occur with epilepsy at rates far higher than in the general population. A Norwegian population-based study among 0‒17-year-olds revealed that 43% of the epileptic children had developmental or psychiatric comorbidities. Complicated epilepsies were associated with higher comorbidity, but uncomplicated cases also had substantial comorbid conditions^2^. Studies evaluating the presence of psychiatric comorbidities in children and adolescents with epilepsy tend to exclude samples indicated for surgery^3^, which was not the case of our work here.

The mechanisms underlying these comorbidities are multiple and, to a certain degree, unclear. For schematic purposes, comorbidities can be explained by the following:

- Shared risk. In this case, there is either a genetic risk for both the epilepsy and the psychiatric disorder and/or an underlying neurological injury or pathological condition that increases the risk of epilepsy and psychopathology. The increased risk of autism in epilepsy, for instance, seems at least in part mediated by common genetic risk^4^.
- The effect of the epileptic activity itself. There is evidence that seizures can lead to building of inadequate cortical networks^5^ and that patients with uncontrolled seizures have a faster decrease in frontocentral and limbic cortices and in thalamic volumes than do patients with good seizure control^6^. Seizures can also be presented as phenotypic copies of psychiatric disorders such as bipolar disorders^7^ or panic disorder^8^.
- The psychosocial burden of epilepsy. Epilepsy may lead to decreased quality of life, which is associated with occurrence of depression^9^. In a study by Novy et al.^10^, high social burden in epilepsy cases explained the association with psychiatric comorbidities.
- The treatment for epilepsy. Use of antiepileptic drugs is associated with a series of cognitive, behavioral and emotional symptoms. For example, topiramate decreases word fluency^11^; levetiracetam is associated with aggressive behavior^12^; and benzodiazepines may be associated with uninhibited behavior^13^^,^^14^. Surgical treatment of epilepsy can have psychiatric consequences too, with development of de novo syndromes, like mania, for example^15^.
- The effect of psychiatric disorders on the seizure threshold. Psychiatric disorders are notably associated with stress. Therefore, even though most antidepressants can lower the seizure threshold, the occurrence of seizures actually might diminish with the treatment for depression^16^^,^^17^^,^^18^, possibly because the amelioration of the depression leads to lower stress levels.
- Others. Psychotropics can alter the seizure threshold. Epilepsy can lead to accidents involving head trauma, which can increase the risk of psychiatric disorders.

Even though the mechanisms underlying comorbidity of epilepsy with psychiatric disorders may be highly complicated, there is significant evidence showing that patients with this comorbidity present associations with lower quality of life, poorer psychosocial adaptation and difficulty in achieving epilepsy control^19^.

In this paper, we aimed to describe the frequency of different psychiatric comorbidities and the use of psychotropic medications in a group of children and adolescents examined in a tertiary epilepsy center who were referred for pediatric psychiatric consultation, including children evaluated for epilepsy surgery.

## METHODS

### Participants

The Ethics Committee of Paulo Niemeyer State Brain Institute approved this retrospective analysis. All medical records of patients who attended at least one consultation at the outpatient child psychiatry clinic of the Epilepsy Center of Paulo Niemeyer State Brain Institute in the city of Rio de Janeiro (Brazil) from January 2014 to July 2016 were reviewed. A single experienced psychiatrist with a PhD who worked exclusively within child and adolescent psychiatry examined the patients.

To make the diagnosis of psychogenic non-epileptic seizure (PNES), we only included patients who had already undergone a video electroencephalogram (v-EEG).

The records of a total of 82 patients were examined. All of these patients had been referred to the child psychiatrist from a neuropediatrician. The patients had to be at most 17 years old at the time of the first interview with the psychiatrist.

### Procedures

The diagnoses first established and medications first and last prescribed were recorded. The patients’ charts were reviewed and diagnoses were ascribed based on the Diagnostic and Statistical Manual of Mental Disorders, 5th Edition (DSM-5)^20^. The child psychiatrist gathered information from a caregiver and/or the patient.

Psychiatric diagnoses were categorized as autism spectrum disorders (ASD), mood disorders, anxiety disorders, disruptive disorders, attention deficit hyperactivity disorder (ADHD), intellectual developmental disorder (IDD), psychosis or others (less frequent diagnoses in this population: specific learning disorders, reactive attachment disorder, encopresis, feeding and eating disorders).

For the purpose of this article, patients were included in the IDD category only if they were not diagnosed with any other psychiatric disorder and had a typical presentation of IDD. The rationale was that we were not endeavoring to extensively analyze the cognitive profile of children with epilepsy. This has been done elsewhere and, since our sample was not extensively evaluated from a neuropsychological point of view, our data would not contribute to the literature. The idea in creating this diagnostic category was to acknowledge that, even though in patients with IDD we also looked for another psychiatric diagnosis that might explain their symptoms, there was a small subset of patients with behavioral symptoms and IDD who did not have other psychiatric diagnoses that would explain their presentation. These are the patients classified as having IDD in this study. Their challenging behavior led to a psychiatric evaluation, but they did not fulfill the criteria for a formal psychiatric diagnosis. Our sample did, of course, have more patients with IDD comorbid with other diagnoses. These, however, were not accounted for here as having IDD.

The patients were divided into groups according to the presence or absence of electroencephalogram changes, among whom epileptiform activity and slow activity on the tracing were highlighted. They were divided according to the location and laterality of the activity in the interictal period. They were classified as having EEG abnormalities in the following regions: temporal, anterior extra-temporal, posterior extra-temporal, hemispheric, para-sagittal or multifocal; or as those who did not present alterations in the examinations.

### Statistical analyses

The Statistical Package for the Social Sciences (SPSS), version 20 for Windows, was used for the analyses. We performed descriptive statistics (means, standard deviations, frequencies and percentages) and comparative analyses (chi-square and Mann-Whitney tests) according to sex, among the diagnoses presented.

## RESULTS

As can be observed in Table 1, sex distribution was similar across the sample and the most frequent age range seen by the psychiatrist was 10-14 years old.

**Table 1:** Clinical and demographic characterization of the sample (n=82).

			n	Percentage (%)
Demographic data	Sex	Female	41	50.0
		Male	41	50.0
	Age range	0‒4 years old	2	2.4
		5‒9 years old	25	30.5
		10‒14 years old	41	50.0
		15‒17 years old	14	17.1
	Age in years	Mean (standard deviation)	10.88 (3.44)	
	City	Rio de Janeiro	50	61.0
		Other cities	32	39.0
Other consultations than neurology and psychiatry	Psychology service		29	35.4
	Neuropsychology service		17	20.7
Psychiatric disorders	Attention deficit/hyperactivity disorder		2	26.8
	Autism spectrum disorders		22	26.8
	Disruptive disorders		20	24.4
	Mood disorders		18	22.0
	Anxiety disorders		17	20.7
	Intellectual disability		13	15.9
	Dissociative/conversive disorders (Psychogenic non-epileptic seizures)		6	7.6
	Other diagnoses		8	9.8
	Psychosis		3	3.7

Autism (26.8%) and ADHD (26.8%) were the most common diagnoses in this sample, but all the other categories (disruptive, mood, anxiety, IDD, dissociative/conversive and psychosis) were present in the sample at frequencies beyond what would be expected from a sample without epilepsy (Table 1).

The medications most commonly prescribed for the patients’ psychiatric conditions were antipsychotics (36.6%) and selective serotonin reuptake inhibitors (25.6%) (Table 2). The antipsychotics prescribed were, in order of frequency: risperidone, aripiprazole, quetiapine, olanzapine and clopixol.

**Table 2:** Medications and examination results of the sample (n=82).

		n	Percentage (%)
Medications	Antipsychotics	30	36.6
	Selective serotonin reuptake inhibitors	21	25.6
	Mood stabilizer	2	2.4
	Stimulant	0	0
	Other	1	1.2
VEEG or EEG location	No alterations	30	36.6
	Anterior extra-temporal	23	28.0
	Temporal	15	18.3
	Posterior extra-temporal	3	3.7
	Hemispheric	3	3.7
	Para-sagittal	3	3.7
	Multiregional	5	6.1
VEEG or EEG lateralization	Left	18	36.0
	Right	11	22.0
	Bilateral	21	42.0
Magnetic resonance imaging	Lesion	25	40.3
	Normal	37	59.7

VEEG: video-eletroencephalogram; EEG: electroencephalogram.

Regarding the type of epilepsy presented, 88.1% of the patients had focal epilepsy, 3.9% had generalized epilepsy and 7.8% had focal and generalized epilepsy, according to the 2017 ILAE classification. Most of the patients (51.3%) had undefined etiology, but 13.1% had genetic causes, 10.5% had encephalomalacia, 7.8% had a tumor, 6.5% had Lennox-Gastaut syndrome, 2.6% had cortical dysplasia, 2.6% had heterotopia, 1.3% had hemimegaloencephaly and 1.3% had juvenile myoclonic epilepsy. Regarding the EEG results, no abnormalities were observed in 36.5% of the patients. Among the changes found, the most common locations were in the frontotemporal (15.3%), temporal (8.2%) and frontal (5.9%) regions (Table 2).

The results from six patients (7.3% of the total sample) with PNES showed that the patients were similarly distributed across the age groups. Regarding comorbidities among the patients with PNES: four patients were diagnosed with a mood disorder (57.1%); two cases were diagnosed with anxiety disorder (28.6%); one patient (14.7%) was diagnosed with an IDD; one patient was diagnosed with ASD (14.7%); no case was diagnosed with disruptive disorders; one case was diagnosed with ADHD (14.7%); and one case was diagnosed with psychosis (14.7%).

## DISCUSSION

Epilepsy is a complex disorder that may occur in consequence of various neurologic injuries, known or unknown. The cause of the epilepsy and the epilepsy itself, directly and indirectly, through the burden of living with epilepsy and its treatments, may have important psychiatric implications. This study aimed to ascertain occurrences of psychiatric disorders in a pediatric sample at a tertiary care epilepsy center in Brazil.

The results indicated that both sexes were affected by comorbid psychiatric diagnoses. Disruptive disorders were significantly more frequent in males, which was not surprising. ADHD (26.8%) and ASD (26.8%) were the most common psychiatric comorbidities; dissociative/conversive disorders (psychogenic non-epileptic seizures) occurred in 7.3% of the sample. The most commonly prescribed drugs were antipsychotics and SSRIs. The high frequency of prescription of antipsychotics might be related to the low availability of behavioral therapy in Brazil, since there is evidence that this kind of treatment is effective for people with IDD and with ASD.

The areas most commonly affected in EEGs relate to the networks involved in psychiatric disorders. Use of an automated diagnostic procedure based on a statistical machine learning methodology using EEGs has been proposed, with promising results^21^. Newson et al.^22^ showed a correlation between frequency bands in EEGs and psychiatric disorders. However, these characteristics were not assessed in our study.

The diagnoses most associated with epilepsy in our sample were ASD and ADHD. These findings regarding comorbidities in epilepsy were also found in previous studies on children with epilepsy^23^^,^^24^. The association between autism and epilepsy is well known. Matsuo et al.^25^ found that 15.2% of their sample of children with epilepsy also had a diagnosis of ASD. On the other hand, autism is also more common in the families of epileptic patients. It has been suggested that the relationship between ASD and epilepsy is bidirectional. Therefore, it is possible that they share common risk factors or heritability^26^. Abnormalities in the GABA system are present both in epilepsy and ASD and in various syndromes in which the prevalence of ASD and epilepsy are increased^27^. Autism is prevalent in patients with epilepsy of many different types, especially when there is co-occurring intellectual disability^28^. The interaction of epilepsy, autism and intellectual disability is further complicated because several characteristics that could serve as signals for the presence of ASD, such as language development delay, are also frequently present in patients with intellectual disability. Accordingly, there are cases of patients with epilepsy and intellectual disability that were spuriously diagnosed with ASD^29^. The second decade of life is a period of increased risk for the development of epilepsy in patients with ASD. However, in approximately 50% of the cases, the epilepsy diagnosis predates the diagnosis of ASD.

ADHD was also a frequent diagnosis. The association between ADHD and epilepsy is more controversial, because some consider that, since epilepsy is frequently associated with cognitive impairments, an additional diagnosis of ADHD should not be granted. A study found that inattentiveness is more severe in patients with epilepsy and ADHD than in children with only ADHD^30^. Accordingly, not all patients with epilepsy have attentional deficits that cause significant impairment. In those who do, appropriate recognition and treatment of ADHD may be useful. The association of stimulants with seizures has hindered the treatment of ADHD in patients with epilepsy, in several cases. This is probably reflected in the low frequency of prescription of stimulants in our sample. It was generally accepted that stimulants could only be used in patients whose epilepsy was under strict control. However, recent evidence has suggested that even patients with refractory epilepsy and ADHD may benefit from the use of stimulants^31^. With the available data, a cautious and individualized approach considering potential risks and benefits is certainly indicated for any patient with epilepsy who presents with significant dysfunctional ADHD symptoms.

As expected, in a center devoted to the treatment of refractory epilepsy, we identified patients diagnosed with dissociative/conversive disorders presenting as PNES (24%), which is expected in adults, according to the previous literature^32^. To our surprise, however, in our sample, there were no patients presenting with both PNES and epilepsy. Previous studies investigating PNES in children and adolescents have also found a notable percentage of individuals with only PNES without epilepsy^33^.

Psychiatric comorbidities in patients with epilepsy are significantly more prevalent than in the general population and have a major impact on the quality of life of patients with epilepsy.
